# Possible NK cell-mediated immune responses against iPSC-derived cells in allogeneic transplantation settings

**DOI:** 10.1186/s41232-020-00150-7

**Published:** 2021-01-06

**Authors:** Kyoko Masuda, Hiroshi Kawamoto

**Affiliations:** Lab of Immunology, Institute for Frontier Life and Medical Sciences, Kyoto University, Kyoto, 606-8507 Japan

**Keywords:** NK cells, Missing-self recognition, KIR-ligand mismatch, Induced pluripotent stem cells (iPSCs), Homo-to-hetero transplantation

## Abstract

In the regenerative medicine field, allogenic transplantation of regenerated tissues has been promoted because autologous transplantation setting is costly and time-consuming to prepare and therefore unsuitable for emergent treatment. To avoid a T cell-mediated immune rejection in the allogenic transplantation setting, induced pluripotent stem cells (iPSCs) derived from different HLA haplotype-homozygous (HLA-homo) donors have been prepared to be used as source of regenerated tissues. However, there still remain immunological issues, even when HLA-homo iPSCs are used. One issue is the immune response against minor histocompatibility antigens expressed on the regenerated tissues, and the other is the immune rejection mediated by NK cells. In this article, we introduce our research on NK cell reactivity against the regenerated tissues in the HLA homo-to-hetero transplantation setting. We further introduce several approaches taken by other groups that address the NK-mediated immune rejection issue.

## Introduction

Embryonic stem cells (ESCs) have long been expected to be the most promising cell source in the regenerative medicine field since human ESCs were established in 1998. The progress of regenerative medicine has given hope to patients who could be cured by replacing damaged tissues or organs with regenerated ones. However, in this case, patients may still face the problem of immune rejection of the regenerated tissues since the transplantation of such ESC-derived regenerated tissue is donor-derived or an “allogenic” transplantation setting. When human induced pluripotent stem cells (iPSCs), which can be established from each patient and are thus “autologous”, were invented in 2007, it was vastly expected that iPSCs can overcome the transplant rejection problem. However, it was not as easy as initially expected; although technically possible, autologous transplantation of iPSC-derived cells or tissues is hardly feasible because the preparation of autologous regenerated tissues is unsuitable for emergency treatment and still require a significant cost. Thus, currently conducted clinical studies are based on transplantation using pre-established allogenic iPSCs derived from healthy donors.

**Fig. 1.**
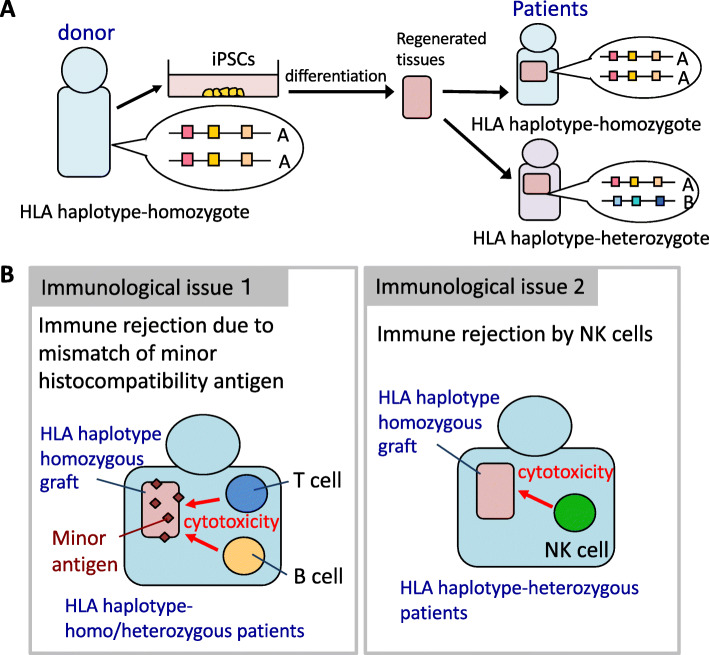
The concept of “iPS Cell Stock Project” and remaining immunological issues. **a** The concept of the “iPS Cell Stock Project”. iPSCs established from monocytes of HLA-homo healthy volunteers can be applied not only to the recipient who has the same HLA in both alleles, but also the one who has it in one allele. No T cell-mediated immune response in the recipient takes place against HLA-homo regenerated graft because the graft has the same HLA as the recipient. **b** Immunological issues in homo-to-hetero transplantation. Issue 1: the mismatch of minor histocompatibility antigens. Minor histocompatibility antigens expressed in grafts do not match to the recipients except in the case of monozygotic twins. Immune rejection may take place when immune cells recognize these antigens in either HLA-homo or HLA-hetero recipients. Issue 2: NK cell-mediated immune reaction. NK cells in the HLA-hetero recipient may recognize HLA-homo regenerated graft and exert cytotoxic activity

For the allogenic use, the iPSCs should be established as highly qualified lines in advance and stocked until use. In Japan, researchers or clinicians can make use of the “iPS Cell Stock Project” in which the iPSC lines that had passed the rigorous quality testing for clinical use have been distributed from the Center for iPS Cell Research and Application (CiRA) at Kyoto University. In this project, iPSCs have been established from monocytes of healthy donors carrying a homozygous human leukocyte antigen (HLA)-haplotype (HLA-homo). Regenerated tissues from HLA-homo iPSCs are assumed to have a lower risk of immune rejection by T cells when these cells are transplanted not only to HLA-homo recipients, but also to HLA haplotype-heterozygous (HLA-hetero) ones [1–3] (Fig. 1a). One iPSC line carrying the most frequent HLA haplotype in the Japanese population, with HLA-A*24:02, HLA-B*52:01, and HLA-DRB1*15:02 in both alleles is estimated to be utilized by 17% of the Japanese population because the allele frequency of this HLA haplotype in Japan is 8.4%. The top 10 or top 70 HLA-homo iPSC lines can cover ~ 50% or ~ 70% of the Japanese population, respectively, has been estimated [2]. Currently, the top 4 HLA-homo iPSC lines are available in this project, covering 34% of the Japanese population.

It is envisioned that regenerated grafts will not be immediately rejected when HLA-homo iPSC-derived regenerated tissues are transplanted to HLA-hetero recipients (homo-to-hetero transplantation) since a T cell-mediated immune reaction could be minimum. However, there are still remaining immunological issues in the aforementioned homo-to-hetero transplantation cases. One is an eminent immune rejection caused by the mismatch of minor histocompatibility antigens, and the other is the NK cell-mediated immune rejection (Fig. 1b). Immune responses against minor histocompatibility antigens, which come about as many proteins contain different amino-acid sequences due to genetic polymorphisms and may take place even in HLA full-matched transplantation except when the donor is the monozygotic twin of the patient. In most cases, grafts may trigger an immune response and will eventually be rejected at some point after the transplantation unless the appropriate immunosuppressant is (lifetime) administered (Fig. 1b, left panel). Moreover, NK cell-based graft rejection will occur in HLA-hetero recipient, the topic on which we mainly focus in this article (Fig. 1b, right panel).

In the field of regenerative medicine, most of investigations have cared the immune reaction mediated by T cells, but not the one mediated by NK cells. We thus investigated to what extent NK cell-mediated immune rejection against transplanted tissues occurs in case of homo-to-hetero transplantation.

## Mechanism of NK cell-mediated immune reaction

NK cells express both activating receptors and inhibitory receptors on their surface. They are in such a balance keeping the NK cells inactive to prevent self-harm. However, when properly licensed NK cells recognize with their activating receptors molecules that are expressed on tumor cells or virally infected cells, they become activated and exert cytotoxic activity against such cells. By contrast, inhibitory receptors prevent NK cell-mediated killing. This review focuses on the inhibitory mechanisms because it will allow to understand the immune response in transplantation immunology. It is known that HLA class I molecules work as ligands of inhibitory receptors of NK cells in human. When NK cells recognize these ligands expressed on somatic cells, the activity of NK cells is suppressed by inhibitory signals and thus NK cells do not show their cytotoxicity. Some infected cells or tumor cells downregulate their HLA expression to avoid the attack by T cells, but it results in inducing the cytotoxic activity of NK cells that sense the lack of HLA expression on the target cells. This sensing mechanism of NK cells is known as “missing-self recognition” [4–8]. This phenomenon was initially reported as a finding that bone marrow cells from the MHC-homo mouse were rejected in MHC-hetero recipient [9], and it was later shown that such rejection was mediated by NK cells [5, 10].

**Fig. 2:**
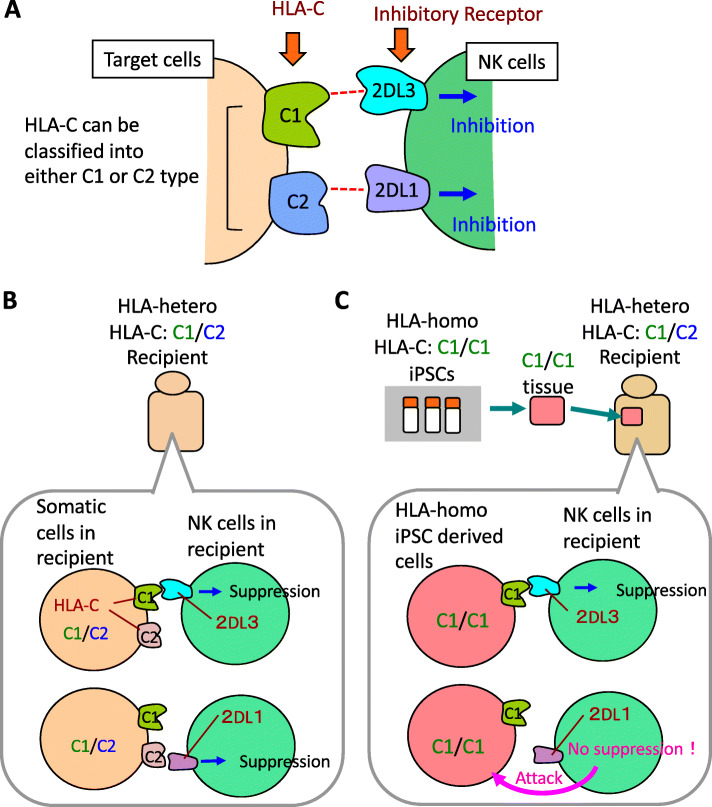
The mechanism of NK cell-based immune responses. **a** Inhibitory receptors and ligands of NK cells. HLA-C molecules function as the major ligands of inhibitory receptors expressed on NK cells. HLA-C molecules can be classified into two types, C1 or C2. The receptor for C1 or C2 is KIR2DL3 (2DL3) or KIR2DL1 (2DL1), respectively. **b** NK cells in recipients carrying HLA-C1/C2 are inhibited by the ligand of either HLA-C1 or HLA-C2 expressed on somatic cells. **c** It is predicted that HLA-hetero C1/C2 recipient NK cells may sense the missing of the C2 molecule on the graft based on the lack of inhibitory signaling when regenerated tissues derived from HLA-homo iPSCs carrying HLA-C1/C1 are transplanted

In human, the major ligands of the NK cell inhibitory receptors are HLA-E, HLA-C, and a part of HLA-B [11]. HLA-E, which is one of the non-classical MHC class I molecules, has limited polymorphism (only 2 alleles) and bind to the CD94/NKG2A receptor on NK cells. Polymorphic HLA-C molecules can be classified into two types, which is determined by the amino acid at position 77 and 80: the C1 epitope has Ser^77^ and Asn^80^, while the C2 epitope has Asn^77^ and Lys^80^. These epitopes bind to NK cell inhibitory receptors that are the member of killer-cell immunoglobuline-like receptor (KIR) family. The C1 epitope is recognized by KIR2DL2 (2DL2) and KIR2DL3 (2DL3), whereas the C2 epitope is recognized by KIR2DL1 (2DL1) (Fig. 2a). HLA-A and HLA-B allotypes that carry the Bw4 motif are recognized by KIR3DL1 (3DL1).

Based on the genotype of HLA-C, graft recipients are divided into 3 types; C1/C1, C1/C2 and C2/C2. In HLA-hetero recipients carrying C1/C2, there exists an NK cell that expresses either 2DL3 or 2DL1 because expression of the KIR molecules is controlled in a random manner, and each type of NK cells is inhibited by the HLA-C1 or HLA-C2 molecule, respectively (Fig. 2b). When regenerated cells from HLA-homo carrying C1/C1 iPSCs are transplanted into HLA-hetero C1/C2 recipients, it is predicted that the recipient’s NK cells may respond by sensing the missing of the C2 molecule on the graft due to the lack of inhibitory signaling (Fig. 2c) and as such participate in graft rejection.

## Immune reaction mediated by NK cells in the homo-to-hetero transplantation setting

**Fig. 3:**
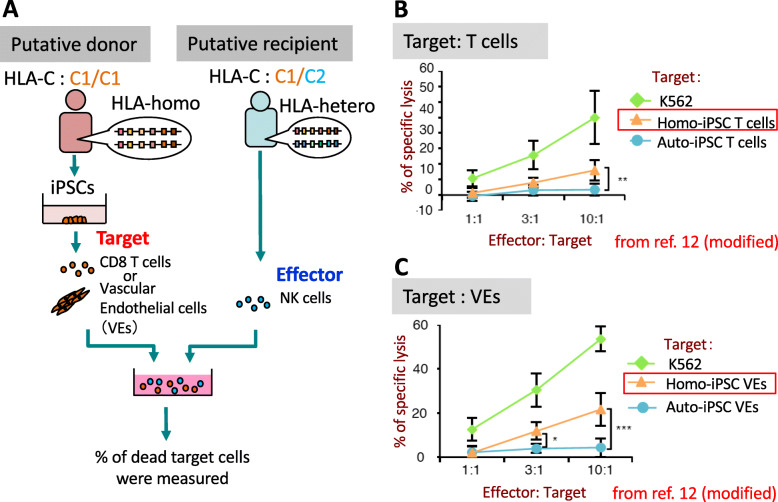
Cytotoxic activity mediated by NK cells in the homo-to-hetero transplantation setting. **a** Schematic illustration of the experimental design. iPSCs were established from an HLA-haplotype homozygous individual as a putative donor and from an HLA-haplotype heterozygous person who carried HLA-C1 and HLA-C2 epitopes as a putative recipient. As target cells, T cells or vascular endothelial cells (VEs) were differentiated from these iPSCs. As effector cells, NK cells collected from peripheral blood mononuclear cells of a putative recipient were used. After effector and target cells were co-cultured for 6 h (T cells) or 18 h (VEs) in different E:T ratio’s, the percentage of dead target cells were measured by Cr^51^-release assay. **b** Cytotoxicity assay of NK cells isolated from the putative recipient against HLA-homo-derived T cells. **p < 0.01, Student’s *t* test. **c** Cytotoxicity assay of NK cells isolated from the putative recipient against HLA-homo-derived VEs. *p < 0.05, ***p < 0.001, Student’s *t* test

We first examined whether NK cells show an immune reaction against the cells regenerated from allogenic HLA-homo iPSCs when KIR ligands are mismatched in a putative “homo-to-hetero” transplantation setting [12]. To this aim, we selected two healthy volunteers as a putative donor and recipient (Fig. 3a). The donor was homozygous for the second most frequent HLA haplotype in the Japanese population (A*33:03, B*44:03, C*14:03, DR*13:02) and was considered as HLA-homo-C1/C1 because HLA-C*14:03 has the C1 epitope. The putative recipient carried the same HLA haplotype on one allele as the donor, and the C2 epitope on the other allele (HLA-hetero-C1/C2). We established iPSCs from the donor (HLA-homo-C1/C1 iPSCs) and the recipient (HLA-hetero-C1/C2 iPSCs). As target cells, T cells or vascular endothelial cells (VEs) were differentiated from these iPSCs using established methods [13, 14]. The reasons to select T cells or VEs as target cell were (1) the plan to use iPSC-derived cytotoxic T lymphocytes for cancer immunotherapy according to the pre-established differentiation protocol [13, 15–17], and (2) VEs will likely be an in vivo target of NK cells due to their direct contact to circulating NK cells [18]. As effector cells, NK cells were collected from the peripheral blood mononuclear cells (PBMCs) of the putative recipient. After these effector and target cells were co-cultured for 6 h in vitro, the percentages of dead cells in the target cell population were determined. Regenerated cells derived from auto-iPSCs (HLA-hetero-C1/C2) or K562 cell line were used as negative and positive control, respectively. NK cells exhibited significant cytotoxic activity against homo-iPSC-derived T cells or VEs at an E:T ratio of 10:1 (Fig. 3b, c), whereas such specific lysis was not detected in target cells from auto-iPSC. These results indicated that NK cells from an HLA-hetero-C1/C2 recipient have the potential to exert cytotoxicity against HLA-homo-C1/C1 iPSCs-derived grafts by the missing-self recognition; i.e., immune rejection mediated by NK cells may take place in a homo-to-hetero transplantation setting in KIR-ligand mismatched cases regarding the C1/C2 epitope.

## A method to avoid NK cell-mediated reaction

**Fig. 4:**
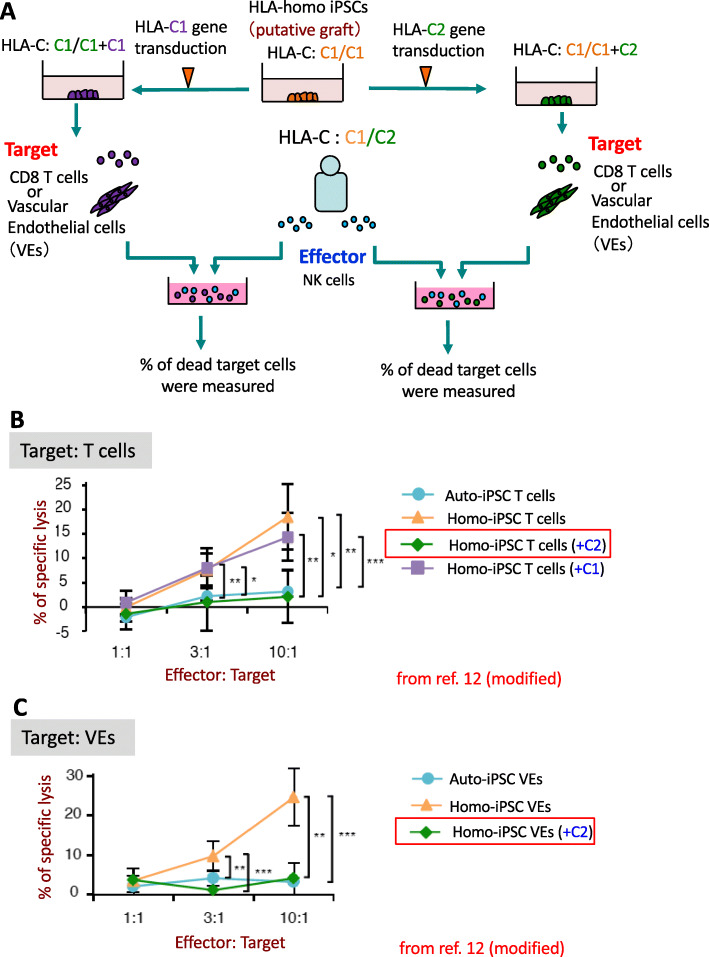
Ectopic expression of the HLA-C2 molecule in regenerated grafts suppresses NK cell alloreactivity. **a** Schematic illustration of the experimental design. HLA-homo-C1/C1 iPSCs were transduced to express type 2 HLA-C allotype that is identical to the putative recipient using a lentiviral system. As negative control, HLA-homo-C1/C1 iPSCs transduced with HLA-C1 gene were also produced. Regenerated T cells or VEs from these iPSCs were used as target cells. After effector and target cells were co-cultured for 6 h in different E:T ratio’s, the percentage of dead target cells were measured by Cr^51^-release assay. **b** Cytotoxic assay of NK cells isolated from the putative recipient against different iPSCs-derived T cells. *p < 0.05, **p < 0.01, ***p < 0.001, Student’s *t* test. **c** Cytotoxic assay of NK cells isolated from the putative recipient against different iPSCs-derived VEs. **p < 0.01, ***p < 0.001, Student’s *t* test

Next, we investigated whether the NK cell-mediated immune reaction can be avoided in the KIR-ligand mismatched situation [12]. As we mentioned above, NK cells were activated by sensing the lack of the C2 epitope on regenerated cells. We hypothesized that the graft rejection reaction could be canceled by overexpressing the KIR ligand with the C2 epitope. Thus, we transduced HLA-homo-C1/C1 iPSCs with the HLA-C2 allele that is identical to the putative recipient (Fig. 4a). As a negative control, we also transduced the same HLA-homo-C1/C1 iPSCs with the HLA-C1 allele. Then, we differentiated these iPSCs into T cells or VEs as target cells and co-cultured them for 6 hours with NK cells obtained from the recipient. Whereas the percentage of specific lysis in target cells derived from iPSCs with the ectopic HLA-C2 expression was almost the same as the one derived from auto-iPSCs, cytotoxic activity was observed against allogenic homo-iPSC-derived regenerated target cells (Fig. 4b, c). These results show that the cytotoxicity against HLA-homo-C1/C1+C2-iPSCs-derived cells can be suppressed by the presence of the C2 epitope on regenerated grafts.

## The frequency of KIR-ligand mismatch in the Japanese population

Our study demonstrated the possibility of immune rejection mediated by NK cells when HLA-homo-iPSC-derived cells are used in the allogenic transplantation setting. Next, we estimated the frequency at which a KIR-ligand mismatch may occur in the Japanese population. The top 4 HLA-haplotype in the Japanese population is shown in Table 1 and all HLA-homo-iPSC lines carrying these haplotypes are C1/C1. Because the allotype frequency of HLA-C1 versus C2 among Japanese is 92.7:7.3 [19], the frequency of a C1/C2 recipient within the HLA-hetero recipients is predicted to be 7.3% in the case of selecting recipients for HLA-homo iPSCs-derived tissues. Thus, the frequency of a KIR-ligand mismatch for HLA-C is rather rare in homo-to-hetero transplantation among Japanese.

**Table 1:**
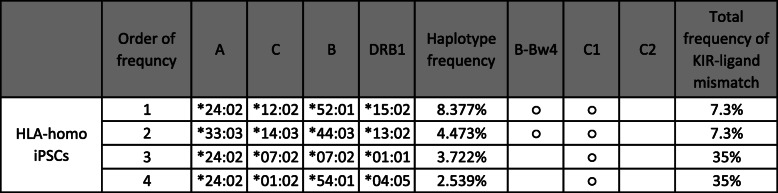
Top 4 HLA haplotype frequencies in the Japanese population and the frequencies of occurrence of a KIR-ligand mismatch

HLA molecules carrying the epitope for ligand of KIR (HLA-B-Bw4, HLA-C1, HLA-C2) are indicated as ○. HLA-homo-iPSC lines of these four haplotypes have been supplied by CiRA Foundation. The allotype frequency of HLA-C1 versus C2 in the Japanese population is 92.7:7.3. When the regenerated tissues from these HLA-homo-iPSCs are transplanted into HLA-hetero recipients, one allele of the recipient must be matched to the HLA-homo iPSCs. As all top 4 HLA haplotype frequencies have the C1 epitope, recipients should have at least a C1 epitope on one allele. On the other hand, because the haplotype of the other allele will be randomly selected, the probability that the recipient has the C1/C1 genotype is 7.3%, because it should be equal to the allotype frequency of the C2 type. The allotype frequency of B-Bw4 in HLA-B is known to be about 30%. A KIR-ligand mismatch will occur when HLA-homo grafts lacking a B-Bw4 ligand are transplanted into an HLA-hetero recipient carrying B-Bw4. Therefore, in the first 2 cases, a B-Bw4 mismatch will not take place. In case of no. 3 and no. 4, one allele of the recipient should be B-Bw-4 negative, because one allele should be the same as the no. 3 or no. 4 haplotype. Because the other haplotype of the recipient is randomly selected, the probability that the recipient has B-Bw4 is the same as the B-Bw4 allotype frequency (30% among Japanese). Thus, in the no. 3 or no. 4 case, the B-Bw4 mismatch occurs with 30% frequency. As the KIR-ligand mismatch regarding C1/C2 or Bw4 takes place in an independent manner, the frequency of KIR-ligand mismatch of at least one mismatch in nos. 3 and 4 case is estimated to be about 35% calculated as [1 − (1 − 0.073) × (1 − 0.3) = 0.3511].

As described above, HLA-B allotypes serving as the Bw4 motif (known as B-Bw4) acting as ligands of 3DL1. The B-Bw4 ligand frequency is about 30% among Japanese. A KIR-ligand mismatch may occur when HLA-homo grafts lacking B-Bw4 ligands are transplanted into HLA-hetero recipients carrying B-Bw4. The third and fourth frequent HLA-homo-iPSCs lack the B-Bw4 ligand. Therefore, a KIR-ligand mismatch regarding the Bw4 motif will take place at substantially high frequency (30%). Taking C1/C2 mismatch also into consideration, total frequency of mismatch for third and fourth iPSCs is estimated to be 35% (Table 1). Although HLA-A*24:02, the most frequent HLA-A allele in the Japanese population, can serve as a Bw4 motif and is categorized as A-Bw4, the KIR-ligand mismatch by A-Bw4 is not considered in this review because it has been reported that the signal is not sufficient to induce the missing-self recognition reaction [20].

## The development of hypoimmunogenic pluripotent stem cells

Several groups have reported the production of hypoimmunogenic pluripotent stem cells, which are designed in such a manner that iPSC-derived cells can avoid the immune rejection caused by not only T cells but also by other immune cells.

**Fig. 5:**
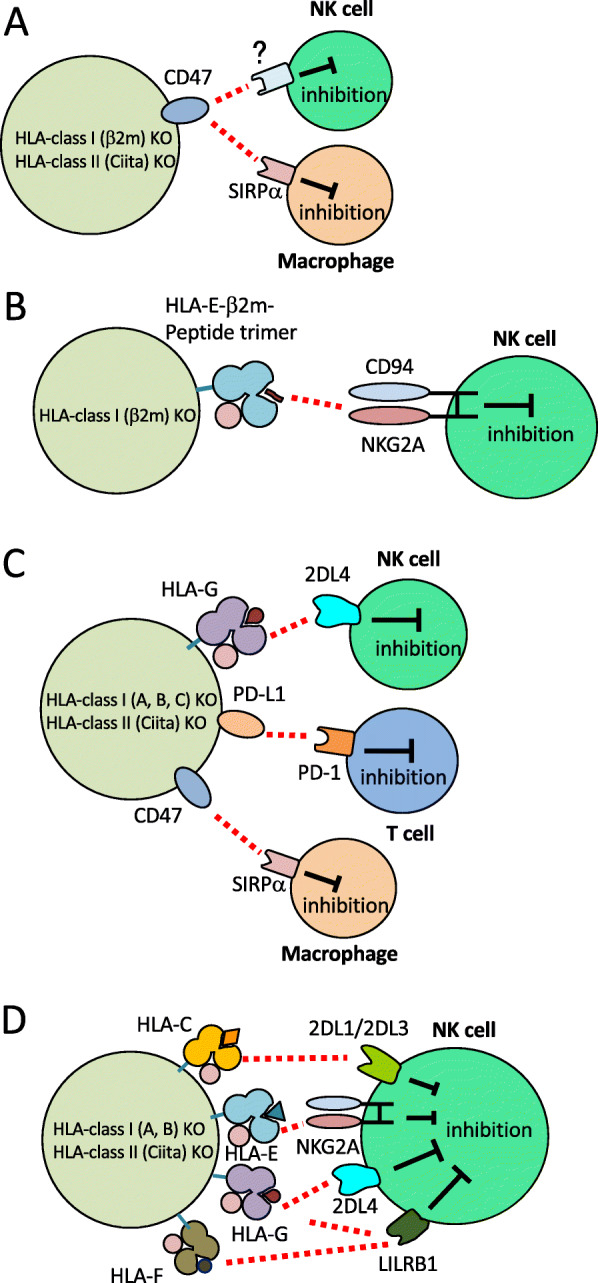
Engineered PSCs designed to prevent the immunological rejection. Based on the disruption of HLA expression on PSCs, CD47 was overexpressed to prevent the engulfment by phagocytosis (**a**), HLA-E molecule was overexpressed (**b**), CD47, PD-L1, and HLA-G were overexpressed (**c**), HLA-C molecules were retained in HLA-homo-iPSCs (**d**)

Apoptotic cells are removed by phagocytic cells, such as macrophages and dendritic cells. During this process, phagocytes recognize the engulfment-inducing signals, such as “find-me” or “eat-me” signals that apoptotic cells express. By contrast, they also can recognize “don’t-eat-me” signals expressed on healthy cells to inhibit the engulfment. One group has reported that the pluripotent stem cells in which CD47, known as one of “don’t-eat-me” signals, was overexpressed following the depletion of HLA molecules [21] (Fig. 5a). In this report, the authors mentioned that overexpression of CD47 in β2 microglobulin (β2m)/class II KO cells impaired NK cell responses. However, in general, it is known that the major effector functions of NK cells are their cytotoxicity and the production of inflammatory cytokines, but not phagocytosis. As the authors did not show the mechanism how the CD47-mediated signal inhibits NK cell activation, it may be possible that impairment of the NK cell-mediated immune response was an indirect effect of CD47 overexpression on the graft.

Another group engineered HLA-E knock-in in human ESCs in which HLA class I has been abrogated by deleting β2m [22] (Fig. 5b). In this report, it was shown that the hematopoietic cells differentiated from these iPSCs were able to avoid the immune response by T cells and NK cells. However, while almost 50% of NK cells express CD94/NKG2A (a major inhibitory receptor for HLA-E), CD94/NKG2A-negative NK cells may attack the regenerated cells by the missing-self recognition. Since NK cells that do not express any inhibitory receptors have not been licensed and are thought to be hypo-responsive, it may be possible that these NKG2A-negative cells contain such hypo-responsive cells. However, a previous report showed that only 20% of NKG2A-negative cells lack expression of all KIRs [23]. Therefore, it is predicted that 80% of NKG2A-negative cells can more or less exert an immune response against HLA class I KO cells expressing HLA-E.

Han et al. have reported the production of PSCs in which HLA class I and II were deleted while HLA-G, CD47, and PD-L1 were knocked-in [24] (Fig. 5c). PD-L1 was knocked-in for the suppression of activated T cells. HLA-G is known to be only expressed on fetus-derived placenta (and some immune privileged tissues) and interacts with maternal NK cells to suppress the immune response [25]. In support of this concept, some groups reported that HLA-G1 positive allografts reduced the NK cell-mediated immune response [26, 27]. However, a contradictory concept regarding the role of HLA-G in placenta has been proposed. KIR2DL4, one of the receptors of HLA-G, is considered as an activating receptor and placental NK cells stimulated by HLA-G promote vascular remodeling of the placenta by producing proangiogenic cytokines [28]. Hence, it is possible that an overexpression of HLA-G leads to enhancement of NK cells mediated responses.

Hotta et al. in Kyoto University have developed HLA-homo-iPSCs in which HLA-A, -B and HLA-II were deleted [29]. These iPSCs retained HLA-C and non-canonical HLA molecules (HLA-E, -F, and -G), which were expected to prevent the immune rejection by NK cells. However, one should still be careful to apply these HLA-C-retaining iPSCs for allogenic transplantation, because the frequency of KIR-ligand mismatch regarding C1/C2 and Bw4 is rather increased (54%) (Table 2) compared when HLA-homo-iPSCs are used (7.3% or 35%) (Table 1).

**Table 2:**
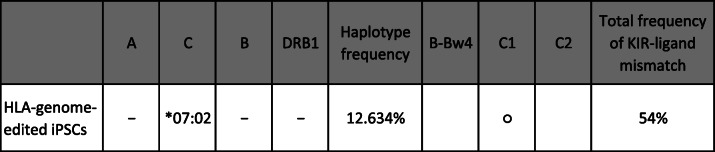
Genome-edited HLA-homo-iPSCs and the frequencies of occurrence of KIR-ligand mismatch

This genome-edited HLA-homo-iPSCs was produced using no. 3 shown in Table 1. Because HLA-A, B, and HLA-II was deleted. The frequency of a KIR-ligand mismatch regarding C1/C2 is 7.3% with the same logic used in Table 1 when the genome-edited HLA-homo-iPSCs carrying C1 epitope was used for the allogenic transplantation. In addition, the frequency of KIR-ligand mismatch regarding Bw4 is increased to 50% calculated as [1 − (1 − 0.3) × (1 − 0.3) = 0.51]. The total frequency of at least one mismatch is estimated to be about 54% calculated as [1 − (1 − 0.073) × (1 − 0.5) = 0.537]

Finally, a general concern can be raised in the strategy using HLA-deleted or HLA-reduced cells. T cells recognize peptides loaded on HLA molecules presented by infected cells and become activated to eliminate those cells. Hence, there are risks that the immune system cannot eliminate transplanted grafts derived from HLA-null PSCs when they are infected by a pathogen or have become malignant. One possible approach to address this issue may be to introduce a suicide gene such as iC9 or HSV-TK into PSCs, by which the transplanted cells can be eliminated upon administration of a certain drugs (like ganciclovir for TK) when these cells become infected or malignant. However, many difficulties remain to be overcome using the suicide system because for instance the introduced gene could be silenced with certain frequency.

## Conclusions

In this review, we have described the possible immune reaction mediated by NK cells in the “homo-to-hetero” transplantation setting. The immune reaction is predictable when the HLA or KIR genotype of recipient is examined. Although several groups propose the use of engineered PSCs lacking or reducing HLA molecule expression, the selection of patients should be carefully done because KIR-ligand match/mismatch cases still remain. In addition, these approaches have not been addressing the issue of immune reactions against minor histocompatibility mismatches. Consequently, immunosuppressants should be given to any patient who undergoes allogenic transplantation. Since it can be expected that the treatment with immuno-suppressive agents also prevents the NK cell-mediated immune reaction, the transplantation across a KIR-ligand mismatch might not be necessarily contraindicated, even though it requires more careful follow-up examinations.

As has been repeatedly mentioned, many clinical applications have been conducted or being planned in Japan using allogenic iPSCs due to the limitation of time and cost to prepare autologous iPSCs from individual patients. Recently, however, the autologous approach is being reconsidered and it has been recently proposed to establish iPSCs and bank them for individuals. Nonetheless, the allogenic transplantation setting is still the current basic strategy in regenerative medicine, and it will be important to develop hypoimmunogenic PSCs to control immune reactions.

## Data Availability

Not applicable

## Abbreviations

ESC: Embryonic stem cell
iPSC: Induced pluripotent stem cell
HLA: Human leukocyte antigen
KIR: Killer-cell immunoglobuline-like receptor
β2m: β2 microglobulin
PBMC: Peripheral blood mononuclear cells
VE cells: Vascular endothelial cells
